# Comment on “Breast Milk: An Optimal Food”

**DOI:** 10.1289/ehp.113-1253739

**Published:** 2005-01

**Authors:** Adriano Cattaneo, Maryse Lehners

**Affiliations:** Unit for Health Services Research and International Health, Child Health Institute, Trieste, Italy, E-mail: cattaneo@burlo.trieste.it; Initiativ Liewensufank, Itzig, Luxemburg, E-mail: maryse.lehners@education.lu

In their editorial “Breast Milk: An Optimal Food,” Pronczuk et al. (2004) stated that “in most cases, mothers can and should be reassured that breast milk is by far the best food to give to their babies,” despite the evidence that “a myriad of potential chemical contaminants … can be detected in breast milk,” mainly because a) levels of environmental contaminants, as determined by subsequent surveys, continue to decrease; b) exposure through breast milk may be less important than exposure in utero; and c) there is little evidence that exposure through breast milk is associated with damage.

We believe that there is probably a fourth good reason in support of their recommendation. There is in fact some evidence that breast-feeding may counteract some of the negative effects of exposure to environmental contaminants in utero.

For example, Boersma and Lanting (2000) showed that at 6 years of age cognitive development is affected by prenatal exposure to polychlorinated biphenyls (PCBs) and dioxins. Breast-fed children, however, when compared to formula-fed children, had an advantage in terms of quality of movements, fluency, and cognitive development tests at 18 and 42 months of age and at 6 years of age, despite a higher PCB exposure from breast milk.

Ribas-Fito et al. (2003), studying a birth cohort of 92 mother–infant pairs highly exposed to organochlorine compounds, found that prenatal exposure was associated with a delay in mental and psychomotor development at 13 months of age and that long-term breast-feeding counterbalanced this damage because it was associated with better performance on both the mental and motor scales compared to short-term or no breast-feeding.

Vreugdenhil et al. (2004) found that children who were breast-fed for at least 16 weeks did not show the delays in development of the central nervous system that are present in children breast-fed for 6–16 weeks or formula-fed, despite a similar prenatal exposure to PCBs.

This evidence is not conclusive (scientific evidence rarely is), but we believe that it should not be omitted in an article on environmental contaminants and breast-feeding.
