# Erratum: Erratum for Johnson et al. [Environ Health Perspect 113:A18 (2005)]

**DOI:** 10.1289/ehp.122-A94

**Published:** 2014-04-01

In the erratum [Environ Health Perspect 113:A18 (2005)] for the article by Johnson et al. [Threshold of Trichloroethylene Contamination in Maternal Drinking Waters Affecting Fetal Heart Development in the Rat. Environ Health Perspect 111:289–292 (2003); doi:10.1289/ehp.5125], the exposure start dates in Table 1 were incorrect for the 2.5-ppb and 250-ppb trichloroethylene (TCE) groups and their concurrent controls. Although the exact dates can no longer be confirmed, the start dates for these three groups occurred in 1994, not 1995.

The authors apologize for the error.

The authors also wish to clarify that all TCE drinking-water exposures lasted throughout gestation, and all of the animal exposure experiments were run with concurrent controls. Rats were ordered based on a 40-animal maximum capacity and were divided among the groups. All groups studied—both exposure groups and concurrent controls—were randomly assigned.

Previously published articles regarding these TCE studies are as follows:

Dawson BV, Johnson PD, Goldberg SJ, Ulreich JB. 1993. Cardiac teratogenesis of halogenated hydrocarbon-contaminated drinking water. J Am Coll Cardiol 21(6):1466–1472.

Johnson PD, Dawson BV, Goldberg SJ. 1998. Cardiac teratogenicity of trichloroethylene metabolites. J Am Coll Cardiol 32(2):540–545.

Johnson PD, Dawson BV, Goldberg SJ. 1998. A review: trichloroethylene metabolites: potential cardiac teratogens. Environ Health Perspect 106(suppl 4):995–999.

Johnson PD, Dawson BV, Goldberg SJ, Mays MZ. 2004. Trichloroethylene: Johnson et al.’s response [Letter]. Environ Health Perspect 112:A608–A609.

